# Retraction: When Words Hurt: Affective Word Use in Daily News Coverage Impacts Mental Health

**DOI:** 10.3389/fpsyg.2020.630044

**Published:** 2020-12-17

**Authors:** Frontiers Editorial Office

The journal retracts the 2 August 2018 article cited above.

This paper reports a study that involved data collected at three time points. The authors recently discovered a clerical error in how the data were merged across time points. Specifically, data collected during the final longitudinal time point (wave 3) were incorrectly assigned to the wrong participants when merging data files. The authors have corrected this error and re-conducted the analyses and the pattern of findings reported in the paper are no longer supported by the analyses.

The authors concur with the retraction and sincerely regret any inconvenience this may have caused to the reviewers, editors, and readers of Frontiers in Psychology.

